# Small extracellular vesicles from menstrual blood-derived mesenchymal stem cells (MenSCs) as a novel therapeutic impetus in regenerative medicine

**DOI:** 10.1186/s13287-021-02511-6

**Published:** 2021-08-03

**Authors:** Lijun Chen, Jingjing Qu, Quanhui Mei, Xin Chen, Yangxin Fang, Lu Chen, Yifei Li, Charlie Xiang

**Affiliations:** 1 State Key Laboratory for Diagnosis and Treatment of Infectious Diseases, National Clinical Research Center for Infectious Diseases, Collaborative Innovation Center for Diagnosis and Treatment of Infectious Diseases, The First Affiliated Hospital, College of Medicine, Zhejiang University, Hangzhou Zhejiang, 310003 People’s Republic of China; 2 Department of Respiratory Disease, Thoracic Disease Centre, The First Affiliated Hospital, College of Medicine, Zhejiang University, Hangzhou Zhejiang, 310003 People’s Republic of China; 3 Department of Intensive Care Unit, The First People’s Hospital of Changde City, Changde, Hunan 415000 People’s Republic of China; 4 Innovative Precision Medicine (IPM) Group, Hangzhou Zhejiang, 311215 People’s Republic of China

**Keywords:** Menstrual blood-derived mesenchymal stem cell (MenSC), Small extracellular vesicle, Exosomes, Cell-free therapy, Regenerative medicine

## Abstract

Menstrual blood-derived mesenchymal stem cells (MenSCs) have great potential in regenerative medicine. MenSC has received increasing attention owing to its impressive therapeutic effects in both preclinical and clinical trials. However, the study of MenSC-derived small extracellular vesicles (EVs) is still in its initial stages, in contrast to some common MSC sources (e.g., bone marrow, umbilical cord, and adipose tissue). We describe the basic characteristics and biological functions of MenSC-derived small EVs. We also demonstrate the therapeutic potential of small EVs in fulminant hepatic failure, myocardial infarction, pulmonary fibrosis, prostate cancer, cutaneous wound, type-1 diabetes mellitus, aged fertility, and potential diseases. Subsequently, novel hotspots with respect to MenSC EV-based therapy are proposed to overcome current challenges. While complexities regarding the therapeutic potential of MenSC EVs continue to be unraveled, advances are rapidly emerging in both basic science and clinical medicine. MenSC EV-based treatment has great potential for treating a series of diseases as a novel therapeutic strategy in regenerative medicine.

**Supplementary Information:**

The online version contains supplementary material available at 10.1186/s13287-021-02511-6.

## Background

Mesenchymal stem cells (MSCs) are heterogeneous subsets of stromal/mesenchymal regenerative cells [1, 2]. They possess powerful self-renewal ability and multi-lineage differentiation potential via symmetric/asymmetric cell division [3–5]. Currently, MSC-based therapy has been diffusely exploited in the treatment of numerous diseases in basic science and clinical medicine [6–12]. Additionally, many clinical trials have proved that MSC infusion is safe and effective at various doses [13–18]. Currently, MSCs can be obtained from almost all parts of tissues/organs, including bone marrow, umbilical cord, adipose tissue, placenta, fetal tissue, Wharton’s jelly, induced pluripotent stem cell (iPSC), embryonic stem cell (ESC), cervical tissue placentae, periodontal ligaments amniotic membrane/fluid, endometrium, lung, liver, dental pulp, peripheral blood, dermal tissues, synovial membranes, and skeletal muscle tissue [19–22]. With the development of personalized medicine, some attractive treatment modalities should be considered to provide precise measures that reflect the underlying biological processes of the complex of diseases in each patient [23–25]. Moreover, with the exception of common sources of MSCs [including bone marrow (BM)-MSCs, umbilical cord (UC)-MSCs, and adipose tissue (AD)-MSCs], other sources should also be considered because these novel sources of MSCs may possess powerful merits in the treatment of corresponding diseases [10, 26–28]. Menstrual blood-derived mesenchymal stem cells (MenSCs) were first found by Meng et al. in 2007 [29]. Since then, MenSC has become a promising therapeutic strategy for the development of effective treatments [30–33]. Compared with other sources of MSCs, MenSCs have several advantages, including abundance, periodic acquisition, non-invasive isolation, high proliferation rate, low immunological rejection, and lack of ethical issues [34–36]. More importantly, MenSCs supply an alternative way that is both painless and free of ethical issues arising from BM-MSCs donations [36]. MenSCs possess a doubling time of approximately 19.4 h, twice as fast as that of BM-MSCs that is estimated at 40–45 h [29]. Menstrual blood in women can be obtained monthly from the age of 20 to 45 years [37–40]. This impressive source is superior to BM-MSCs, AD-MSCs, and UC-MSCs. Although extensive progress has been made in deciphering the immunosuppression/immunoregulation of MSCs, the study on the immunoregulation of MenSCs is still in its infancy [34]. It is only known that MenSCs do not express MHC-II. Therefore, the slow progress in the immunoregulation of MenSCs greatly limits the application of MenSCs. Based on these advantages, MenSCs have been continuously reported for treating various diseases in both basic science and clinical medicine [37, 40–43].

An increasing number of studies have demonstrated that the therapeutic benefits of MSCs are principally mediated via paracrine roles, through the secretion of growth factors, chemokines, and cytokines rather than their differential abilities or cellular replacements [5, 10, 44–53]. Therefore, researchers are increasingly interested in the therapeutic value of MSC-derived bioactive molecules, especially the secretome and extracellular vesicles (EVs), which are considered the key components of paracrine effect in the treatment of MSC-based therapy [54–56]. Furthermore, researchers have shown that MSC-conditioned medium induced repair of injured tissues in several animal models [47, 57]. Compared with MSC-based therapy, MSC EV-based therapy is highly recommended because it is less likely to trigger an immune-repulsion response and is safe to the host, not causing ethical problems [58, 59]. In addition, EVs have different routes of injections, including intranasal, oral, intravenous, intraperitoneal, and subcutaneous [60–64]. Thus, MenSC-derived EVs offer important application advantages. In this review, we systematically discuss the current progress of MenSC-derived EVs with regard to the identification of components, functions, and therapeutic potential in treating a series of diseases. Moreover, we highlight current challenges and promising perspectives of MenSC-derived EVs in regenerative medicine to guide future clinical applications.

## The basic characteristics and biological functions of MSC-derived small EVs

EVs are generally released from the endosomal compartments, present in almost all body fluids, and released by all types of cells [65, 66]. They are involved in multiple pathological processes with cell-to-cell communication monitoring, showing promising therapeutic potential in different diseases [67–70]. Classically, EVs are generally divided into exosomes, microvesicles, and apoptotic bodies, based on their sizes, origins, biogenesis, and cargo: (1) exosomes, diameter of 30–150 nm, fused with the cell membrane through multivesicular bodies to deliver into the extracellular body; (2) microvesicles, diameter of 50–1000 nm, derived from the direct budding of the plasma membrane; (3) apoptotic bodies, a diameter of 100–5000 nm, displaying wide distributions [71–73]. Their biological functions are shown in Table 1.

**Table 1:** Biological functions of extracellular vesicles (EVs) in body fluids

EVs functions	Exosomes	Microvesicles	Apoptotic bodies
**Origin**	Endosomal multivesicular bodies	Cell surface	Apoptotic cell surfaces
**Generation**	Intracellular vesicle traffic	Plasma membrane	Plasma membrane
**Size**	30–150 nm	50–1000 nm	100–5000 nm
**Markers**	Tetraspanins (CD9/63/81), Alix, HSP70/90, flotillin, TSG101, clathrin, GM130, MHC	Annexin V, selectins, integrins, flotillin-2, CD40, metalloproteinases	Histones, Annexin V
**Cargos**	Proteins, lipids, mRNA, miRNA, DNA, carbohydrates	Proteins, mRNA, miRNA	Proteins, mRNA, miRNA, fragment of DNA

As consensus has not yet emerged on specific markers of EV subtypes, it is hard to distinguish exosomes or microvesicles; therefore, MSC exosomes or microvesicles are referred to as MSC-derived small EVs, following the classical references [74–77]. Small EVs consist of various biomolecules, such as regulatory proteins, small peptides, lipids, and some genetic materials (including mRNA, small RNA, long non-coding RNA, genomic DNA, complementary DNA, and mitochondrial DNA), which are delivered to a spectrum of recipient cell types [78–81]. Over the past decade, small EVs have emerged as major mediators of cell-free therapy and are a promising tool for a variety of diseases. In view of their exceptionally broad biological functions, small EVs can stimulate targeting cells, transfer membrane receptors, deliver proteins or genetic information, and eventually cause epigenetic differences in recipient cells [82–85]. In addition to cell communication, it is increasingly evident that small EVs have an important function in regulating different physiological processes, such as cell maintenance, immune surveillance, cell migration, tissue repair, glycometabolic regulation, cell differentiation, cancer therapy, hematopoietic engraftment, blood coagulation, and angiogenesis [86–92]. Thus, small EVs offer a unique platform for the development of a novel class of therapeutics for the treatment of various diseases.

Generally, MSC-derived small EVs share an evolutionarily conserved set of molecules, including membrane transport and fusion proteins (GTPases, annexins, and flotillin), heat shock protein (HSP) family (HSP20, HSP27, HSP40, HSP60, HSP70, and HSP90), tetraspanins (CD9, CD63, and CD81), multivesicular body biogenesis [ALG-2-interacting protein-X (Alix) and TSG101], as well as some lipid-related proteins and phospholipases [93–96]. The therapeutic potential of MSC-derived small EVs is usually elicited by delivering biologically relevant proteins and RNAs to recipient cells [97]. Accumulating evidence shows that MSC-derived small EVs are successfully applied as therapy of several disease models [98–105]. Recently, small EVs have been reported as the principal therapeutic agents with regenerative capabilities and immunomodulatory functions of MSC secretions [75, 80, 106, 107]. To date, MSC-derived small EVs have been isolated from a series of sources, including human/mouse/rat/canine/pig bone marrow [108–112], human/mouse/rat/canine/equine/mini-pig adipose tissue [112–117], mouse cardiac tissue [118], and human umbilical cord [119], ESC [120], iPSC [121], menstrual blood [122], Wharton’s jelly [123], placental and fetal tissue [124, 125], dental pulp [126], gastric cancer tissue [127], synovial membrane [128], corneal [129], fetal liver [130], oral mucosa [131], and amniotic fluid [132]. Detailed information on reports of MSC-derived small EVs from different sources is presented in Table 2. Although an increasing number of sources of MSCs are being evaluated for their role in exosomes, the underlying mechanism and appropriate source need to be further explored.

**Table 2:** Different sources of MSC-derived small EVs

Source of MSC-derived small EVs	Year	Reference
Human ESC	2010	[120]
Rat BM	2012	[108]
Mouse BM	2012	[109]
Human BM	2013	[110]
Human AD	2013	[113]
Human UC	2013	[119]
Human placental	2013	[124]
Human Wharton’s jelly	2013	[123]
Human gastric cancer tissue	2014	[127]
Fetal tissue	2014	[125]
Dental pulp	2015	[126]
Human iPSC	2015	[121]
Rat AD	2016	[114]
Mouse AD	2016	[115]
Human menstrual blood	2016	[122]
Mini-pig AD	2016	[116]
Human amniotic fluid	2016	[132]
Human synovial membrane	2016	[128]
Human Corneal	2018	[129]
Pig BM	2018	[111]
Mouse cardiac	2018	[118]
Human fetal liver	2019	[130]
Human oral mucosal	2019	[131]
Equine AD	2019	[117]
Canine BM and AD	2019	[112]

Notes: *ESC*, embryonic stem cell; *BM*, bone marrow; *AD*, adipose; *UC*, umbilical cord; *iPSC*, induced pluripotent stem cell; *AM*, amniotic membrane

**Fig. 1:**
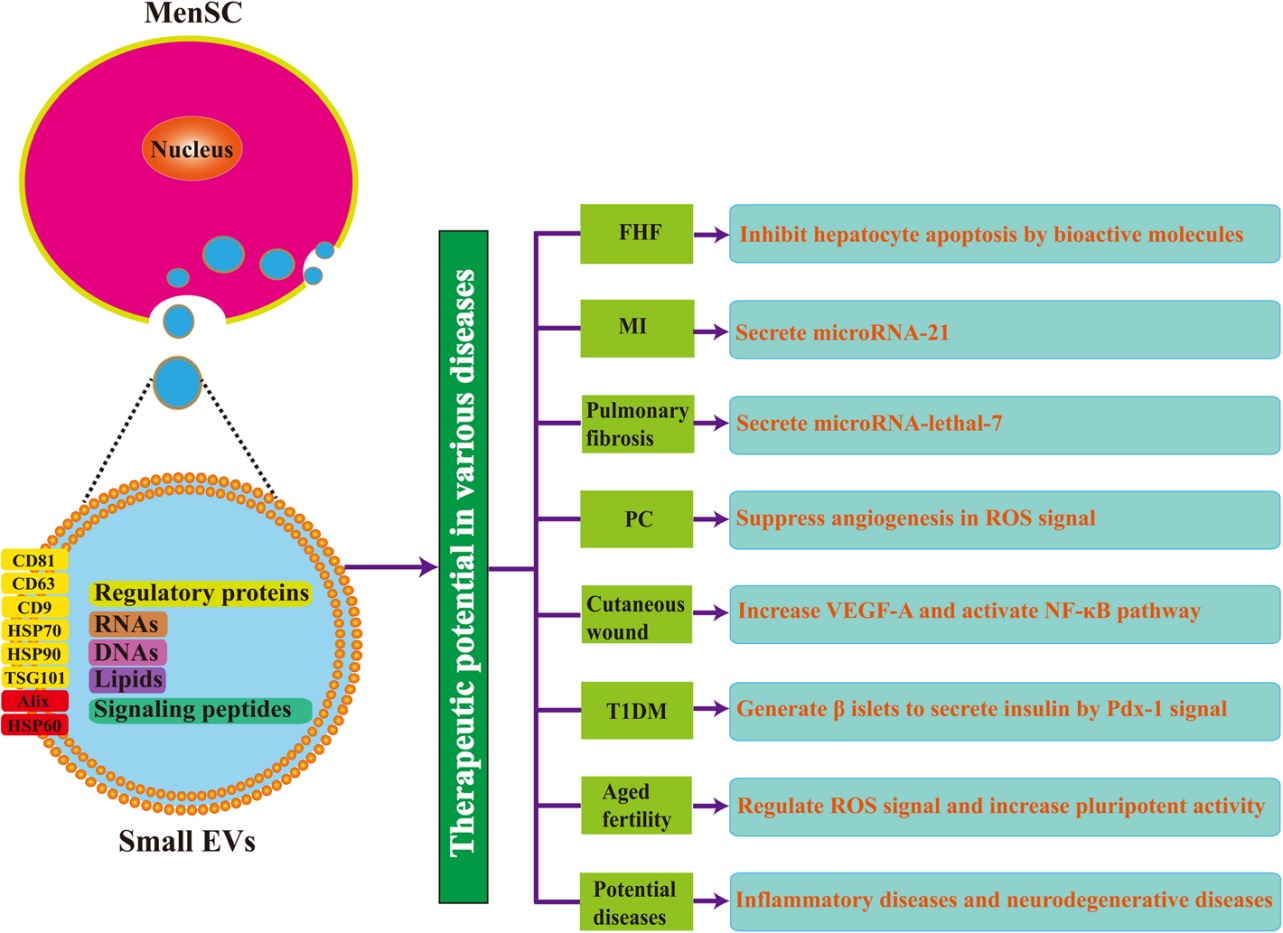
Identification of MenSC-derived small EVs and their therapeutic potentials for tissue repair in various diseases. Small EVs from MenSCs consist of regulatory proteins, RNAs, and DNAs, lipids, and siginaling peptides promoting regenerative repair of wounded cells and tissues. MenSC-derived small EVs are positive for the expression of CD9, CD63, CD81, HSP70, HSP90, and TSG101, and they are negative for Rab5 and calnexin. The expression of HSP60 and Alix, which are positive for universal MSC-derived small EVs, need to be recognized for further verification. The therapeutic potential of MenSC-derived small EVs in various diseases, including fulminant hepatic failure (FHF; via inhibition of hepatocyte apoptosis by bioactive molecules), myocardial infarction (MI, via secreted microRNA-21), pulmonary fibrosis (via secreted microRNA-lethal-7), prostate cancer (PC; via suppression of angiogenesis by ROS signaling), cutaneous wound (via increase in VEGF-A and activation of NF-κB pathway), type-1 diabetes mellitus (T1DM; via generation of β islets to secrete insulin by Pdx-1 signaling), aged fertility (via regulation of ROS signaling and increase in pluripotent activity), and some potential diseases (such as inflammatory and neurodegenerative diseases)

MenSC-derived small EVs were first reported by Lopez-Verrilli et al. in 2016 [122], and the authors revealed that MenSC-derived small EVs promote axonal regeneration after nerve injury in the central and peripheral nervous system. Previous studies showed that MenSC-derived small EVs express CD63 and TSG101 [133, 134], and other researchers further discovered that MenSC-derived small EVs present CD9, CD81, HSP70, and HSP90 [122, 135–140]. Additionally, MenSC-derived small EVs do not express Rab5 or calnexin [122, 136, 138, 140]. Thus, protein markers of MenSC-derived small EVs should include CD9, CD63, CD81, HSP70, HSP90, and TSG101 and exclude Rab5 and calnexin (Fig. 1). Although these markers are commonly studied, some other molecules (such as HSP60 and Alix) still need to be recognized in accordance with universal MSC-derived small EVs [94, 106, 141]. Moreover, serving as a unique tissue type source of MSCs, some representative markers from MenSC-derived small EVs should be identified to represent the specific source of MSCs. Although research on MenSC-derived small EVs is relatively new compared to common sources of MSCs, the basic definition and identification of MenSC-derived small EVs should be established for future research.

## Therapeutic potential of MenSC-derived small EVs in treating various diseases

In contrast to numerous studies on small EVs from common sources of MSCs (such as BM-MSCs, AD-MSCs, and UC-MSCs), the research on the therapeutic potential and underlying mechanisms of MenSC-derived small EVs are still in an initial stage. In this context, although the therapeutic effect of MenSC has been demonstrated since 2007 [29], the study on MenSC-derived small EVs was first reported in 2016 [122]. Owing to the superiority of MenSC gradually emerging in recent years [31, 33, 34, 142], studies on MenSC-derived small EVs have great potential and profound significance in regenerative medicine, as shown in Fig. 1.

### MenSC-derived small EVs for fulminant hepatic failure (FHF)

FHF, also termed acute liver failure (ALF), is a progressive, life-threatening, and sharp pathological reaction characterized by hepatic dysfunction [143]. Currently, orthotopic liver transplantation (OLT) is the most effective treatment for FHF. However, because of the shortage of donor organs, high transplantation costs, and accurate expertise needed for the surgery, an increasing number of researchers are seeking other available methods to treat FHF. It has been verified that MenSC-derived small EVs have an effect in suppressing hepatocyte apoptosis in a D-galactosamine (D-GalN)/lipopolysaccharide (LPS)-induced FHF model in mice [133]; also, the expression of tumor necrosis factor-α (TNF-α), interleukin (IL)-6, and IL-1β was evidently reduced in co-culture of alpha mouse liver 12 (AML12) hepatocytes with MenSC-derived small EVs in vitro. Additionally, the effective bioactive molecules for ameliorating FHF were mainly mediated by MenSC exosomes of angiopoietin-2, intercellular adhesion molecule-1 (ICAM-1), anexelekto, IL-6, osteoprotegerin, IL-8, insulin-like growth factor-binding protein-6 (IGFBP-6), and angiogenin [133].

### MenSC-derived small EVs for myocardial infarction

Myocardial infarction (MI), a type of coronary artery disease, is caused by apoptosis of cardiomyocytes due to excessive ischemic conditions [144]. Because MI has a long-term undiscovered period, it usually leads to severe hemodynamic deterioration and sudden death. Thus, a novel therapeutic strategy is required to treat MI. Wang et al. discovered that transplantation of MenSC-derived small EVs significantly improved cardiac function in infarcted rat hearts [134]. The authors further found that microRNA (miR)-21 secreted from MenSC-derived small EVs played a dominant role in improving MI in the animals. The exosomal miR array showed that miR-21 targets phosphatase and tensin homolog (PTEN) and the downstream molecule of AKT/PKB (protein kinase B) to trigger signal cascades. This result showed that MenSC-derived small EVs ameliorate the damaged cardiac function in MI primarily through the paracrine function on excretive miR-21.

### MenSC-derived small EVs for pulmonary fibrosis

Pulmonary fibrosis is a chronic problem that is of widespread concern [145]. Lung transplantation is currently the optimal treatment for this disease, but it is limited by the lack of donors; thus, an alternative method is required for pulmonary fibrosis treatment. Sun et al. verified that transplantation of MenSC-derived small EVs significantly ameliorated bleomycin-induced pulmonary fibrosis by repairing alveolar epithelial cell injury in a mouse model in vivo and in vitro [146]. Further investigation revealed that miR lethal-7 (let-7) of MenSC-derived small EVs enhanced the ability of lectin-like oxidized low-density lipoprotein receptor-1 (LOX-1) to inhibit the activation of reactive oxygen species (ROS) and mitochondrial-DNA damage by regulating NOD-, LRR-, and pyrin domain-containing protein 3 (NLRP3) signaling pathway. Thus, targeting miRs (such as let-7) of MenSC-derived small EVs is a promising approach for the treatment of pulmonary fibrosis.

### MenSC-derived small EVs for prostate cancer

Prostate cancer (PC) is an epithelial malignancy that occurs in the prostate and is the third-leading cause of cancer mortality in men [147]. Although comprehensive treatments (such as surgery radiotherapy, endocrine therapy, and radiation) are used in PC patients, the practical effect is still far away from curing the disease [148]. Some researchers have found that MSC-derived small EVs have the ability to ameliorate the tumor microenvironment by limiting tumor growth, angiogenesis, and metastasis, mainly targeting fibroblasts, endothelial cells, and immune cells [149]. Recently, Alcayaga-Miranda et al. proved that MenSC-derived small EVs significantly inhibited tumor angiogenesis in the PC3 tumors model in mice [135]. Moreover, the antitumor effect contributed to a decrease in vascular density and tumor hemoglobin content. MenSC-derived small EVs inhibited the secretion of vascular endothelial growth factor (VEGF) and hypoxia-inducible factor-1α (HIF-1α) and reduced the activity of nuclear factor kappa B (NF-κB). The authors further proved that MenSC-derived small EVs lowered reactive oxygen species (ROS) production in PC3 cells. In this context, a previous study showed that ROS regulates angiogenesis and tumor development through HIF-1α and VEGF in PC3 cells [150]. Therefore, these results indicate that MenSC-derived small EVs act as a blocker of tumor-induced PC angiogenesis by suppressing tumor-induced angiogenesis via a ROS-dependent mechanism.

### MenSC-derived small EVs for cutaneous wounds

Cutaneous wounds commonly occur via loss of structures and appendages by externally acute stimulants (such as extensive burns, scalds, trauma, or diabetic ulcers) that induce chronic wounds/scars [151]. MSC-derived small EVs have therapeutic potential in cutaneous repair and regeneration [152]. Dalirfardouei et al. showed that MenSC-derived small EVs significantly reduced cutaneous damage in diabetic foot ulcers in mice [140]. Wound healing mainly contributes to the polarization of M1-M2 macrophages by increasing VEGF-A to promote angiogenesis and activating NF-κB to alleviate local inflammation.

### MenSC-derived small EVs for type-1 diabetes mellitus (T1DM)

T1DM is caused by multiple factors that lead to an increase in blood glucose concentration and a severe decrease in insulin secretion [153]. Currently, transplantation of islets is the most effective treatment; however, it is restricted owing to the lack of sufficient pancreatic donors. The therapeutic potential of MenSCs for treating T1DM has been verified [154]. Mahdipour et al. demonstrated that MenSC-derived small EVs have a therapeutic function, improving T1DM in rats [138]. The authors also found that administration of MenSC-derived small EVs improved the regenerative capacity of β islets and facilitated the production of insulin through the pancreatic and duodenal homeobox 1 (Pdx-1) signaling cascade.

### MenSC-derived small EVs for aged fertility

With social and financial pressure, an increasing number of women have postponed motherhood after the age of thirty-five. However, because of the poor quality and insufficient quantity of oocytes, the overall pregnancy rate and fertility level is low [155, 156]. Therefore, improving the quality of oocytes or activating aging oocytes is a viable route to improve the fertility of aged women [157]. Different sources of MSC-derived small EVs play a vital role in improving ovarian insufficiency age-related fertility [139]. Moreover, EVs can be used to improve the quality of embryos during assisted reproduction [157]. Marinaro et al. found that MenSC-derived small EVs increased embryo quality and quantity by regulating antioxidant enzymes and increasing pluripotent activity in an aged mouse model [139]. Additionally, MenSC-derived small EVs showed the ability to increase the developmental level of in vitro fertilization-derived embryos via an ROS-dependent approach in aged female mice [137]. Based on the proteomics analysis of murine blastocysts, some core genes related to cellular response to oxidative stress (*Gpx1* and *Sod1*), metabolism (*Acaca* and *Gapdh*), placentation (*Pgf*, *VEGF-A*), and trophectoderm/inner cell mass formation (*Pou5f1* and *Sox2*) are the most likely candidates for improving embryo quality and quantity [137, 139]. Other researchers found that miR-17-5P, miR-223-3P, miR-146a-5p, and miR-21-5p from UC-MSC-derived small EVs are possible contributors to improving ovarian insufficiency or age-related fertility [158–160]. Additionally, Zhao et al. revealed that increased expression of integrin-β3, leukemia inhibitory factor, and VEGF in AD-MSC-derived small EVs may promote endometrial regeneration and fertility restoration [161].

### MenSC-derived small EVs for potential diseases

Although many studies have focused on the mutual effect between MenSC-derived small EVs and specific disease models, the study of the interaction between MenSC-derived small EVs and pro-inflammatory conditions also provides a direction for regenerative medicine. Marinaro et al. used a comprehensive proteomics and transcriptomics analysis and found that some functionally immunomodulatory proteins [including colony-stimulating factor-1, PYCARD (PYD and CARD domain), and endoplasmic reticulum aminopeptidase 1 (ERAP1)] regulate immune responses in interferon (IFN)-γ primed MenSC-derived small EVs [162]. Thus, MenSC-derived small EVs have a promising immunomodulatory potential for treating inflammation-related diseases in future studies. Additionally, Lopez-Verrilli et al. found that MenSC-derived small EVs effectively enhanced the growth of primary neuronal cells [122]. The authors showed that MenSC-derived small EVs have superior potential when compared with MSC-derived small EVs from other sources (including bone marrow, umbilical cord, and chorion) in neurodegenerative diseases.

## Current challenges of MenSC-derived small EVs for tissue repair

Although MenSC-derived small EVs have been described in several studies, the effective elements of small MenSC-derived EVs remain a mystery. Small EVs contain bioactive molecules that affect the characteristics of target cells [82, 98]. Additionally, the involvement of miRNAs in the cellular and molecular mechanisms of MenSC-derived small EVs is of great importance, but to date, only a few miRNAs (miR-21and let-7) have been explored [134, 146]. In fact, MenSC expresses octamer-binding transcription factor 4 (OCT-4), which is a marker of ESC [154], a distinct marker compared with other sources of MSCs. Research on MenSC-derived small EVs is relatively limited compared with MSC-derived small EVs from other sources (such as bone marrow, adipose tissue, and umbilical cord). Currently, the similarity of therapeutic mechanisms between MenSC-derived and other sources of small EVs is mainly due to the secretion of effective bioactive molecules and production of miRNAs [163]. The miR-21, miR-27a, miR-196a, and miR-206 are abundant in EVs from BM-MSCs and are responsible for pro-regenerative and immunomodulatory effects [164–166]; miR-20, miR-21, miR-23a, miR-125b, miR-326, and miR-145 are profuse in EVs from UC-MSCs and are responsible for mediation of apoptosis, regulation of autophagy, inhibition of neddylation, and suppression of myofibroblast differentiation [167–169]; let-7, miR148a, miR378, and miR532-5p are abundant in EVs from AD-MSCs and are responsible for angiogenesis, cellular transport, apoptosis, and proteolysis [170, 171]; and let-7 and miR21 are abundant in EVs from MenSCs and are responsible for regulating mitochondrial-DNA damage and enhancing cell survival rate [134, 146]. Several studies explored MSC-derived small EVs signaling pathways [64, 160, 172, 173], supporting that a thorough database of small EVs from MenSCs is needed to further assess their therapeutic potential. Additionally, current studies about small EVs from MenSCs are relatively few and most of them are preliminary, the further in-depth comparisons are necessary between MenSC-derived and other sources of MSC-derived small EVs. And distinct bioactive elements and special signaling pathways from MenSC-derived small EVs are needed to be explored in the future.

Determining the optimal dose and appropriate time points for the administration of small EVs without adverse effects are vital issues. The quality control of MenSC-derived small EVs is an important factor, an indispensable link in the process for the final approval of MenSC-derived small EV therapy. The quality of small EVs mainly includes characteristics, purity, efficacy, safety, and stability based on a large amount of data to establish the standards of consistency and stability. Although MenSC is a heterogeneous cell population, as a minimum standard catalog, it must follow the current guidelines of the International Society for Cellular Therapy [174]. Different methods to separate and quantify MenSC-derived small EVs with different identification standards may cause controversy and reduce reliability in experimental conclusions. It is difficult to analyze and compare exosomes from different sources because the corresponding contents are also discrepant. Therefore, establishing a unified standard of MenSC-derived small EVs will facilitate their clinical application.

The long-term effect of MenSC-derived small EVs is a vital issue that needs to be addressed in regenerative medicine. There are few studies concerning the sustained therapeutic effects. Current purification and enrichment strategies (including ultracentrifugal collection, tandem filtration, and polyethylene glycol precipitation) of MSC-derived small EVs originate from the manufacturing methods of viruses or viral-like particles. The stabilization of the purity and physiological function of MenSC-derived small EVs remains a problem. Therefore, if any viral-related products (including lentiviral and adenoviral vectors of gene editing) are present in the conditioned medium or recipient cell, they will be enriched in the final exosome extraction, which is a potential risk for safe use. In addition, small EVs contain abundant small RNAs. These small RNAs may increase the instability of nucleic acid chains or cause structural changes in partial tissues along with some complications [175, 176]. Therefore, before MenSC-derived small EVs are applied in clinical medicine, more studies are required with a large number of basic medicine and clinical trials to assess their long-term safety.

## Future perspectives of MenSC-derived small EVs in regenerative medicine

As there is great potential for the clinical application of MenSC-derived small EVs, novel strategies should be developed to expedite this process. Future perspectives of MenSC-derived small EVs with regard to regenerative medicine will be devoted to the aspects subsequently described (Fig. S1).

### Engineered MenSC-derived small EVs

Currently, genome editing is a novel technology widely applied in genetic modifications, functional genomics, transcriptional regulations, and stem-cell therapies. With the rapid development of CRISPR/Cas9, engineered MSC-derived small EVs are a powerful tool [94, 177, 178]. This modification can be achieved by overexpressing proteins or modifying miRs in MSCs to achieve changes in exosomes [179]. These engineered MSC-derived small EVs have a higher therapeutic potential than the initial MSC-derived small EVs. This has been proven for small EVs from miRs (including miR-92a-3p, miR-133b, miR-181-5p, miR-22-3p, miR-31, miR-466, and miR-584)-engineered MSCs [180–186]. Additionally, small EVs from proteins (including SDF-1, TRAIL, TIMP2, P53, IDO1, and PEDF)-engineered MSCs also improved the treatment outcome in regenerative medicine [187–192]. Owing to the therapeutic potential of MenSC-derived small EVs in several diseases, some engineered small EVs of MenSC are establishing a foundation for clinical trials and clinical medicine. As there are only sporadic studies on miR-21 engineered MenSC-derived small EVs in treating MI [134], more engineered MenSC-derived small EVs should be explored.

### Hypoxia-treated MenSC-derived small EVs

Hypoxia is an important feature of various tumors. It can maintain the survival of tumor cells and has a strong correlation with tumor invasion and poor prognosis [193]. Hypoxic cells undergo extensive intracellular molecular and metabolic regulation to create a tumor microenvironment that is conducive to their survival and growth. Cells secrete various cytokines, exosomes, proteins, nucleic acids, and lipids during hypoxia. In fact, hypoxia-treated MSC-derived exosomes have a better effect in treating diseases. Small EVs from hypoxia-treated human AD-MSCs have a high ability to increase angiogenesis through VEGF/VEGF-receptor and protein kinase A (PKA) signaling pathways [194, 195]. Zhu et al. discovered that BM-MSC-derived small EVs effectively protected the cardiac function through miR-125b in a hypoxia-induced MI mouse model [196]. Cheng et al. found that BM-MSC-derived small EVs restrained apoptosis to improve myocyte protection in a hypoxia-challenged MI rat model, partially owing to exosomes containing miR-210 [197]. Thus, hypoxia-treated MenSC-derived small EVs could be a strong candidate for enhancing the cardiac function.

### MenSC-derived small EVs combined with targeting drugs

Small EVs have a series of advantages as drug carriers, such as unique structure and physicochemical properties, effective cell access, low immunogenicity and toxicity, and natural capacity to cross organism barriers [198, 199]. Additionally, MSC-derived small EVs can deliver drugs to recipient cells in a highly selective manner [98, 200]. In other words, MSC-derived small EVs are an ideal delivery system for small molecular drugs. Chang et al. found that AD-MSC-derived small EVs combined with 50 mg/kg/day melatonin improved acute inflammatory colitis in a rat model [201]. Kalimuthu et al. verified that paclitaxel (25, 50, and 100 mg/mL) mixed with BM-MSC-derived small EVs were more powerful than single BM-MSC-derived small EVs in inhibiting breast cancer [202]. The authors revealed that the loading efficiency was 38.9, 76.1, and 74.22 ng/mg for 25, 50, and 100 mg/mL of paclitaxel, respectively. Currently, targeting drugs vary with specificity and uptake efficiency of recipient cells; thus, further investigation is needed to confirm the optimum dose of each qualified drug. We believe that targeting drugs combined with MenSC-derived small EVs is promising to exert a stronger role than that of MenSC-derived small EVs alone.

### MenSC-derived small EVs from three-dimensional cultures

Three-dimensional (3D) tissue-specific cultures have been a powerful tool in disease therapy in recent years, and a large number of studies have been conducted on various diseases [203]. Currently, 3D structures can be derived from pluripotent stem cells (including ESCs and iPSCs) or adult stem cells (including epithelial cells and MSCs [204, 205]. 3D culture can provide researchers with precise control over spatial heterogeneity within the tumor microenvironment by spatially depositing predefined biobanks that contain multiple stem-cell types, biochemical factors, and ECMs [206, 207]. Kim et al. found that 3D-cultured MSCs significantly enhanced the secretion efficiency of exosomes and their production [208]. Furthermore, exosomes from 3D-cultured BM-MSCs [209] and UC-MSCs [210] showed a powerful regeneration capacity. Although 3D culture from single-cultured MSCs has not been systematically reported, small EVs from 3D-cultured MenSCs would produce abundant bioactive molecules to meet the dose requirements for clinical medicine.

### MenSC-derived small EVs for cancer immunotherapy

The successful application of immune checkpoint inhibitors of cytotoxic T lymphocyte antigen-4, programmed cell death protein 1 (PD-1), and programmed cell death protein ligand 1 (PD-L1) in various diseases has attracted interest in the field of immunotherapy, especially cancer immunotherapy [211–213]. The underlying function of small EVs has been explored in cancer immunotherapy as a novel therapeutic strategy [214, 215]. The immune-modulation of MSC-derived small EVs has been applied, for example, to improve skin regeneration [216], protect against hearing loss [217], prevent inflammation, or induce remyelination in multiple sclerosis [218], graft-versus-host disease [219], and asthma [220]. Marinaro et al. revealed that MenSC-derived small EVs exert immunomodulatory effects in the treatment of inflammatory conditions by immunomodulatory proteins and several miRNAs using proteomics and genomics analyses [162]. Thus, MenSC-derived small EVs may be a competitive candidate for future cancer immunotherapy owing to their outstanding immunomodulatory role.

### MenSC-derived small EVs immobilized in hydrogel

The use of chemical materials with biological functions may be an interesting candidate to transfer MSC-derived small EVs [221]. Biomaterials can provide matrix interaction, enhancing the transmission effect of MSC-derived small EVs and affect secretion characteristics through signal transmission from outside to inside. Currently, well-defined synthetic hydrogels are promising carriers for the delivery of stem cells [222, 223]. Shi et al. found that the combination of human gingival exocrine MSCs and hydrogel can effectively alleviate skin wound healing in diabetic rats by improving collagen epithelialization, deposition, and remodeling and increase angiogenesis and neuron growth [224]. Zhang et al. verified that chitosan hydrogel combined with MSC-derived small EVs significantly enhanced the therapeutic roles of hindlimb ischemia, via firefly luciferase imaging of angiogenesis [225]. Zhao et al. found that chitosan hydrogel-encapsulated MSC-derived small EVs significantly prolonged the aging of skin processes by improving the function of old dermal fibroblasts [226]. Li et al. established a system for human MSC-derived small EVs immobilized in an exosome peptide-modified adhesive hydrogel (Exo-pGel), which effectively migrated to the spinal cord injury microenvironment and exerted evident nerve recovery and urinary tissue preservation through relieving inflammation and oxidation [227]. Thus, the function of MenSC-derived small EVs may effectively enhance immobilization in hydrogels, and this may be a promising strategy in future regenerative medicine.

## Conclusions

**Fig. 2:**
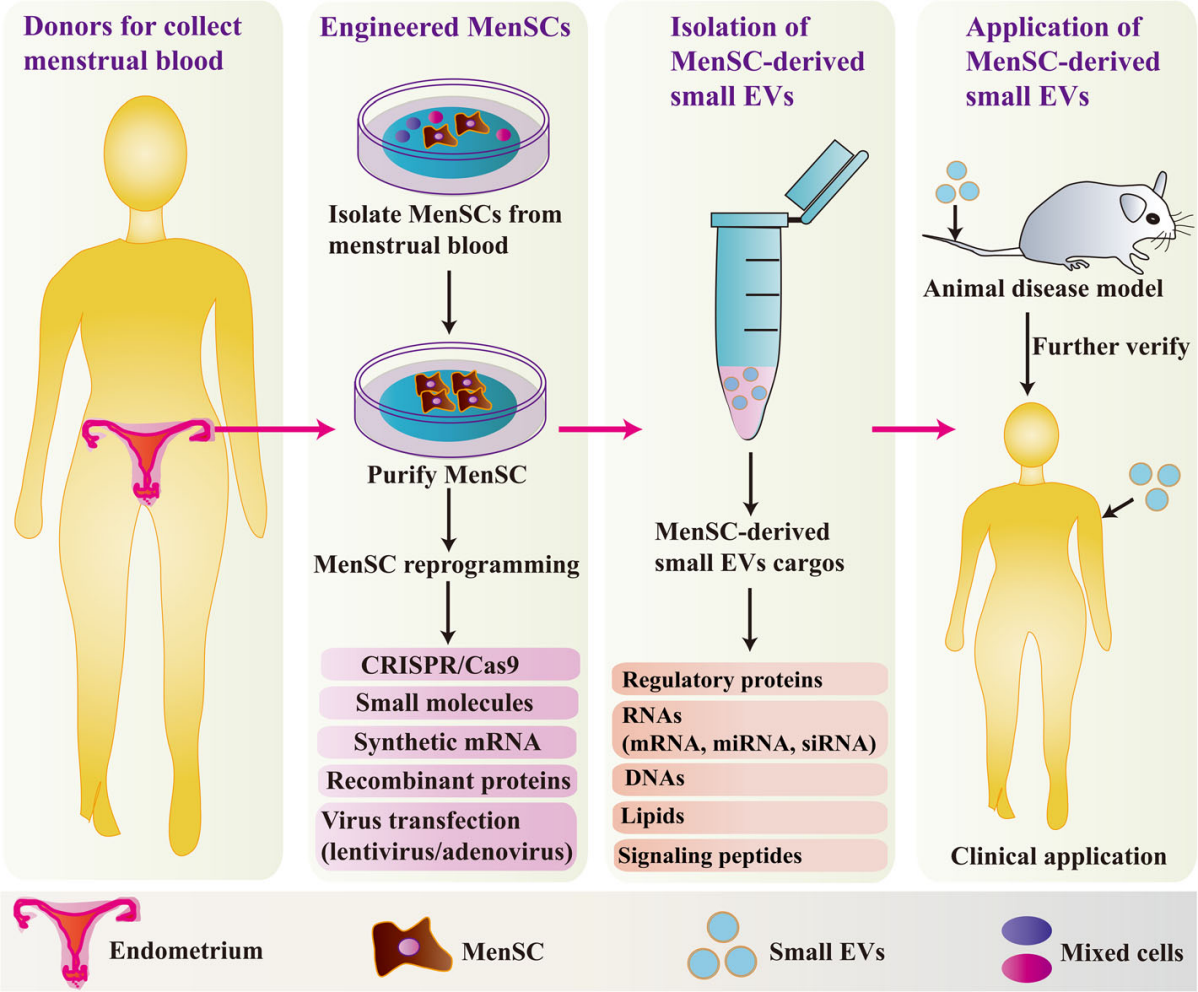
The strategy for developing clinical applications of MenSC-derived small EVs. Tissue donors should be selected and examined for MenSC-derived small EVs production. Donors can be autologous or allogeneic obtained from menstrual blood. MenSCs modification by bioengineering [CRISPR/Cas9, small molecules, synthetic mRNA, virus transfection (lentivirus/adenovirus), recombinant proteins] may be considered to improve therapeutic efficacy of MenSC-derived small EVs. Therapeutic effects of engineered MenSC may be further improved by encapsulating miRNA or siRNA in them. The application of MenSC-derived small EVs first evaluated the safety and effectiveness through an animal disease model. Then, MenSC-derived small EVs were employed to treat a variety of diseases in the clinic

MenSC-derived small EVs deliver a large amount of regulatory proteins and mRNAs to improve the regenerative repair of wounded cells and tissues. While complexities about their therapeutic potential continue to be unraveled, advances are continuously found in both basic science and clinical medicine. Novel techniques (including engineered molecules, hypoxia-treated conditions, targeting drugs, 3D culture, cancer immunotherapy, and hydrogel) with respect to MenSC-derived small EVs may further promote translational medicine. Additionally, a strategy for developing the clinical use of MenSC-derived small EVs was proposed (Fig. 2). Rapid progress in separation techniques and combinations are available in MSC-derived small EVs, as important sources of MSC, MenSC, and MenSC-derived small EVs should be explored in the future. Additionally, the function of MenSC-derived small EVs also needs to be investigated for further comparisons with other sources of MSC-derived small EVs. In summary, although more research is needed, MenSC-derived small EV-based therapy has great potential for treating various diseases in regenerative medicine.

## Supplementary Information

**Additional file 1: Fig. S1.** Perspectives of MenSC-derived small EVs in regenerative medicine. The electron microscopy of MenSC-derived small EVs are labelled with a yellow arrow (the scale is 200 nm). As the great potential of the clinical application of MenSC-derived small EVs, novel strategies with regard to future perspectives of MenSC-derived small EVs are shown in: (1) engineered MenSC-derived small EVs, which is contributed by gene editing (such as CRISPR/Cas9, overexpression and RNA interference); (2) hypoxia-treated MenSC-derived small EVs, which improves the microenvironment conducive to their survival and growth; (3) MenSC-derived small EVs combined with targeting drug, which enhances the delivering efficiency of drugs to recipient cells; (4) 3D-culture of MenSC-derived small EVs, which increases the secreting efficiency of small EVs; (5) MenSC-derived small EVs for cancer immunotherapy, which enhances the immunomodulatory role; (6) MenSC-derived small EVs immobilized in hydrogel, which enhances the transmission effect.

## Data Availability

Please contact the corresponding author for data requests.

## Abbreviations

AD: Adipose tissue
ALF: Acute liver failure
Alix: ALG-2-interacting protein-X
AML12: Alpha mouse liver 12
BM: Bone marrow
ESC: Embryonic stem cell
ERAP1: Endoplasmic reticulum aminopeptidase 1
EV: Extracellular vesicle
Exo-pGel: Exosome peptide-modified adhesive hydrogel
FHF: Fulminant hepatic failure
HIF-1α: Hypoxia-inducible factor-1α
HSP: Heat shock protein
ICAM-1: Intercellular adhesion molecule-1
IFN: Interferon
IGFBP-6: Insulin-like growth factor-binding protein-6
IL: Interleukin
iPSC: Induced pluripotent stem cell
let-7: Lethal-7
LPS: Lipopolysaccharide
LOX-1: Lectin-like oxidized low-density lipoprotein receptor-1
MenSC: Menstrual blood-derived mesenchymal stem cell
MI: Myocardial infarction
miR: MicroRNA
MSC: Mesenchymal stem cell
NF-κB: Nuclear factor kappa B
NLRP: NOD-, LRR-, and pyrin domain-containing protein 3
OLT: Orthotopic liver transplantation
PC: Prostate cancer
PD-1: Programmed cell death protein 1
PD-L1: Programmed cell death protein ligand 1
Pdx-1: Pancreatic and duodenal homeobox 1
PK: Protein kinase
PTEN: Phosphatase and tensin homolog
ROS: Reactive oxygen species
TNF-α: Tumor necrosis factor-α
T1DM: Type-1 diabetes mellitus
UC: Umbilical cord
VEGF: Vascular endothelial growth factor
3D: Three-dimensional
